# Supplementary material for: Assessment of Motor Abilities and Physical Fitness in Youth in the Context of Talent Identification—OSF Test
Source: Int J Environ Res Public Health. 2022 Nov 1;19(21):14303. doi: 10.3390/ijerph192114303 (PMC9655008; doi:10.3390/ijerph192114303)
Supplement: Supplementary file 1 [file ijerph-19-14303-s001.zip › S2 - Supplementary Materials 2 OSF tables of normalised values.pdf]

Tables of normalised values

Total

Table S1. Values for setting norms in general

	3x10m run - result	Standing broad jump - result	1 kg medicine ball throw - result	4-minute run - result	Total
$\bar{x}$	8.538	1.756	7.131	814.443	
**SD**	0.840	0.287	2.067	133.358	
**0%**	-3.135	-4.029	-2.918	-4.908	-12.176
**1%**	-1.948	-2.217	-1.803	-2.239	-7.308
**2%**	-1.769	-1.938	-1.610	-1.942	-6.411
**3%**	-1.663	-1.764	-1.513	-1.719	-5.820
**4%**	-1.591	-1.624	-1.416	-1.570	-5.442
**5%**	-1.473	-1.590	-1.367	-1.570	-5.140
**6%**	-1.425	-1.485	-1.270	-1.422	-4.838
**7%**	-1.354	-1.415	-1.222	-1.347	-4.588
**8%**	-1.294	-1.311	-1.173	-1.273	-4.380
**9%**	-1.235	-1.241	-1.125	-1.199	-4.177
**10%**	-1.199	-1.241	-1.125	-1.199	-4.001
**11%**	-1.116	-1.241	-1.052	-1.124	-3.815
**12%**	-1.116	-1.137	-1.028	-1.124	-3.642
**13%**	-1.057	-1.067	-1.028	-1.050	-3.510
**14%**	-0.997	-1.067	-0.979	-0.976	-3.378
**15%**	-0.986	-0.962	-0.931	-0.976	-3.230
**16%**	-0.914	-0.893	-0.931	-0.901	-3.108
**17%**	-0.879	-0.893	-0.883	-0.901	-2.986
**18%**	-0.879	-0.893	-0.858	-0.827	-2.872
**19%**	-0.831	-0.893	-0.834	-0.827	-2.750
**20%**	-0.784	-0.823	-0.786	-0.790	-2.637
**21%**	-0.760	-0.788	-0.786	-0.753	-2.521
**22%**	-0.760	-0.718	-0.786	-0.678	-2.415
**23%**	-0.736	-0.718	-0.737	-0.678	-2.317
**24%**	-0.689	-0.718	-0.689	-0.678	-2.225
**25%**	-0.653	-0.649	-0.689	-0.641	-2.125
**26%**	-0.641	-0.614	-0.640	-0.604	-2.028
**27%**	-0.641	-0.544	-0.640	-0.567	-1.937
**28%**	-0.617	-0.544	-0.592	-0.530	-1.851
**29%**	-0.582	-0.544	-0.574	-0.530	-1.759
**30%**	-0.534	-0.544	-0.543	-0.455	-1.663
**31%**	-0.522	-0.544	-0.543	-0.455	-1.573
**32%**	-0.522	-0.544	-0.543	-0.455	-1.483

	3x10m run - result	Standing broad jump - result	1 kg medicine ball throw - result	4-minute run - result	Total
$\bar{x}$	8.538	1.756	7.131	814.443	
**SD**	0.840	0.287	2.067	133.358	
**33%**	-0.499	-0.474	-0.519	-0.418	-1.386
**34%**	-0.463	-0.439	-0.495	-0.381	-1.307
**35%**	-0.427	-0.370	-0.446	-0.381	-1.219
**36%**	-0.404	-0.370	-0.446	-0.322	-1.138
**37%**	-0.404	-0.370	-0.446	-0.307	-1.060
**38%**	-0.392	-0.370	-0.398	-0.232	-0.985
**39%**	-0.368	-0.300	-0.374	-0.232	-0.907
**40%**	-0.332	-0.265	-0.349	-0.232	-0.840
**41%**	-0.285	-0.230	-0.340	-0.232	-0.764
**42%**	-0.285	-0.195	-0.301	-0.195	-0.690
**43%**	-0.285	-0.195	-0.301	-0.158	-0.609
**44%**	-0.249	-0.195	-0.277	-0.158	-0.530
**45%**	-0.214	-0.195	-0.253	-0.084	-0.448
**46%**	-0.166	-0.195	-0.214	-0.084	-0.382
**47%**	-0.166	-0.161	-0.204	-0.084	-0.310
**48%**	-0.166	-0.126	-0.156	-0.084	-0.237
**49%**	-0.130	-0.056	-0.156	-0.047	-0.161
**50%**	-0.107	-0.021	-0.131	-0.009	-0.090
**51%**	-0.047	-0.021	-0.107	-0.009	-0.013
**52%**	-0.047	-0.021	-0.059	0.028	0.057
**53%**	-0.047	0.014	-0.059	0.065	0.136
**54%**	-0.012	0.049	-0.059	0.065	0.213
**55%**	0.024	0.083	-0.059	0.080	0.292
**56%**	0.072	0.153	-0.010	0.139	0.372
**57%**	0.072	0.153	-0.010	0.139	0.450
**58%**	0.083	0.153	0.038	0.214	0.531
**59%**	0.131	0.153	0.038	0.214	0.610
**60%**	0.167	0.153	0.087	0.214	0.687
**61%**	0.190	0.153	0.111	0.214	0.768
**62%**	0.190	0.188	0.135	0.288	0.853
**63%**	0.226	0.223	0.184	0.288	0.928
**64%**	0.273	0.293	0.184	0.288	1.019
**65%**	0.309	0.327	0.232	0.325	1.099
**66%**	0.309	0.327	0.232	0.362	1.192
**67%**	0.321	0.327	0.281	0.362	1.276
**68%**	0.368	0.397	0.329	0.437	1.358
**69%**	0.428	0.432	0.329	0.437	1.450
**70%**	0.428	0.502	0.377	0.496	1.549

	3x10m run - result	Standing broad jump - result	1 kg medicine ball throw - result	4-minute run - result	Total
$\bar{x}$	8.538	1.756	7.131	814.443	
**SD**	0.840	0.287	2.067	133.358	
**71%**	0.428	0.502	0.426	0.511	1.637
**72%**	0.487	0.502	0.426	0.511	1.726
**73%**	0.547	0.502	0.426	0.585	1.826
**74%**	0.547	0.502	0.474	0.585	1.924
**75%**	0.547	0.571	0.523	0.660	2.024
**76%**	0.606	0.641	0.547	0.660	2.119
**77%**	0.665	0.676	0.620	0.660	2.226
**78%**	0.665	0.711	0.668	0.660	2.341
**79%**	0.689	0.780	0.668	0.734	2.465
**80%**	0.744	0.815	0.717	0.793	2.579
**81%**	0.784	0.850	0.789	0.808	2.704
**82%**	0.808	0.850	0.838	0.883	2.833
**83%**	0.879	0.850	0.896	0.920	3.003
**84%**	0.903	0.920	0.911	0.957	3.136
**85%**	0.962	0.990	0.959	1.031	3.288
**86%**	1.022	1.024	1.007	1.031	3.435
**87%**	1.069	1.129	1.104	1.106	3.594
**88%**	1.140	1.199	1.153	1.173	3.793
**89%**	1.200	1.199	1.250	1.254	4.000
**90%**	1.259	1.268	1.347	1.284	4.196
**91%**	1.378	1.373	1.395	1.361	4.407
**92%**	1.497	1.478	1.444	1.403	4.660
**93%**	1.568	1.547	1.589	1.477	4.942
**94%**	1.651	1.617	1.686	1.589	5.251
**95%**	1.734	1.727	1.880	1.775	5.644
**96%**	1.877	1.896	2.049	1.849	6.104
**97%**	2.067	2.070	2.364	2.146	6.619
**98%**	2.328	2.244	2.607	2.295	7.380
**99%**	2.835	2.628	3.188	2.667	8.618
**100%**	8.979	4.684	5.272	4.005	14.612

Girls

Table S2. Values for setting norms for girls

	3x10m run - result	Standing broad jump - result	1 kg medicine ball throw - result	4-minute run - result	Total
$\bar{x}$	8.653	1.712	6.793	794.003	
SD	0.821	0.257	1.814	123.486	
0%	-3.081	-4.311	-3.136	-5.167	-12.303
1%	-1.951	-2.373	-1.922	-2.350	-7.493
2%	-1.769	-2.022	-1.702	-1.986	-6.612
3%	-1.647	-1.985	-1.591	-1.784	-5.971
4%	-1.526	-1.791	-1.536	-1.623	-5.565
5%	-1.453	-1.598	-1.426	-1.542	-5.210
6%	-1.404	-1.598	-1.371	-1.502	-4.970
7%	-1.293	-1.481	-1.316	-1.381	-4.721
8%	-1.282	-1.404	-1.260	-1.300	-4.470
9%	-1.185	-1.326	-1.205	-1.219	-4.262
10%	-1.161	-1.210	-1.150	-1.179	-4.100
11%	-1.100	-1.210	-1.095	-1.139	-3.892
12%	-1.039	-1.210	-1.040	-1.098	-3.713
13%	-1.039	-1.171	-0.985	-1.058	-3.548
14%	-1.003	-1.055	-0.985	-1.026	-3.379
15%	-0.942	-1.016	-0.985	-0.977	-3.250
16%	-0.918	-1.016	-0.929	-0.896	-3.129
17%	-0.918	-0.939	-0.929	-0.896	-2.991
18%	-0.869	-0.861	-0.874	-0.856	-2.873
19%	-0.821	-0.822	-0.874	-0.816	-2.749
20%	-0.796	-0.822	-0.819	-0.775	-2.638
21%	-0.796	-0.822	-0.819	-0.735	-2.524
22%	-0.784	-0.822	-0.764	-0.735	-2.408
23%	-0.736	-0.745	-0.764	-0.695	-2.302
24%	-0.687	-0.706	-0.709	-0.654	-2.187
25%	-0.675	-0.629	-0.709	-0.573	-2.087
26%	-0.675	-0.629	-0.654	-0.573	-1.983
27%	-0.651	-0.629	-0.654	-0.573	-1.898
28%	-0.614	-0.590	-0.598	-0.573	-1.803
29%	-0.578	-0.512	-0.598	-0.533	-1.698
30%	-0.553	-0.474	-0.543	-0.493	-1.602
31%	-0.553	-0.435	-0.543	-0.452	-1.510
32%	-0.541	-0.435	-0.516	-0.412	-1.423
33%	-0.517	-0.435	-0.488	-0.412	-1.336
34%	-0.480	-0.435	-0.433	-0.331	-1.251
35%	-0.432	-0.435	-0.433	-0.331	-1.150
36%	-0.432	-0.396	-0.433	-0.331	-1.060

	3x10m run - result	Standing broad jump - result	1 kg medicine ball throw - result	4-minute run - result	Total
$\bar{x}$	8.653	1.712	6.793	794.003	
**SD**	0.821	0.257	1.814	123.486	
**37%**	-0.432	-0.357	-0.433	-0.331	-0.966
**38%**	-0.408	-0.318	-0.378	-0.251	-0.878
**39%**	-0.371	-0.241	-0.378	-0.251	-0.804
**40%**	-0.322	-0.241	-0.323	-0.251	-0.713
**41%**	-0.310	-0.241	-0.323	-0.194	-0.636
**42%**	-0.310	-0.241	-0.323	-0.170	-0.556
**43%**	-0.286	-0.163	-0.268	-0.129	-0.477
**44%**	-0.250	-0.125	-0.268	-0.089	-0.407
**45%**	-0.213	-0.086	-0.212	-0.089	-0.327
**46%**	-0.189	-0.047	-0.212	-0.089	-0.255
**47%**	-0.189	-0.047	-0.157	-0.081	-0.183
**48%**	-0.177	-0.047	-0.157	-0.008	-0.112
**49%**	-0.140	-0.047	-0.157	-0.008	-0.020
**50%**	-0.104	-0.047	-0.102	0.032	0.063
**51%**	-0.067	-0.008	-0.102	0.072	0.156
**52%**	-0.067	0.030	-0.047	0.072	0.226
**53%**	-0.043	0.069	-0.047	0.072	0.303
**54%**	-0.007	0.108	0.008	0.072	0.381
**55%**	0.030	0.147	0.008	0.113	0.450
**56%**	0.054	0.147	0.063	0.153	0.542
**57%**	0.054	0.147	0.063	0.153	0.613
**58%**	0.091	0.185	0.119	0.153	0.687
**59%**	0.127	0.263	0.119	0.234	0.765
**60%**	0.176	0.302	0.119	0.234	0.858
**61%**	0.176	0.340	0.119	0.234	0.948
**62%**	0.176	0.340	0.174	0.274	1.032
**63%**	0.224	0.340	0.174	0.315	1.111
**64%**	0.273	0.340	0.229	0.355	1.199
**65%**	0.297	0.340	0.229	0.395	1.291
**66%**	0.297	0.340	0.284	0.395	1.366
**67%**	0.309	0.418	0.312	0.395	1.449
**68%**	0.370	0.457	0.339	0.420	1.538
**69%**	0.419	0.496	0.394	0.476	1.619
**70%**	0.419	0.534	0.394	0.476	1.715
**71%**	0.419	0.534	0.450	0.476	1.796
**72%**	0.461	0.573	0.450	0.541	1.902
**73%**	0.516	0.612	0.505	0.557	2.005
**74%**	0.540	0.651	0.560	0.597	2.090
**75%**	0.540	0.728	0.560	0.638	2.189

	3x10m run - result	Standing broad jump - result	1 kg medicine ball throw - result	4-minute run - result	Total
$\bar{x}$	8.653	1.712	6.793	794.003	
SD	0.821	0.257	1.814	123.486	
76%	0.577	0.728	0.615	0.678	2.309
77%	0.638	0.728	0.670	0.718	2.402
78%	0.662	0.728	0.670	0.718	2.497
79%	0.686	0.728	0.670	0.799	2.597
80%	0.747	0.806	0.753	0.799	2.700
81%	0.783	0.883	0.781	0.880	2.820
82%	0.820	0.922	0.836	0.880	2.928
83%	0.893	0.922	0.891	0.880	3.031
84%	0.905	0.999	0.946	0.920	3.153
85%	0.978	1.077	1.001	0.960	3.283
86%	1.026	1.116	1.056	1.041	3.412
87%	1.087	1.116	1.111	1.082	3.561
88%	1.148	1.116	1.222	1.162	3.720
89%	1.245	1.193	1.222	1.203	3.903
90%	1.306	1.271	1.277	1.283	4.048
91%	1.391	1.310	1.387	1.364	4.215
92%	1.500	1.426	1.498	1.405	4.401
93%	1.573	1.503	1.608	1.526	4.612
94%	1.634	1.503	1.718	1.606	4.910
95%	1.756	1.620	1.773	1.687	5.178
96%	1.877	1.697	1.939	1.768	5.465
97%	2.096	1.891	2.215	2.010	5.887
98%	2.363	2.046	2.435	2.292	6.430
99%	2.849	2.279	2.877	2.680	7.358
100%	7.589	4.217	4.586	4.109	11.112

Boys

Table S3. Values for setting norms for boys

	3x10m run - result	Standing broad jump - result	1 kg medicine ball throw - result	4-minute run - result	Total
$\bar{x}$	8.375	1.817	7.604	843.027	
SD	0.836	0.313	2.293	141.127	
0%	-2.955	-3.258	-2.532	-4.122	-12.189
1%	-1.881	-2.041	-1.745	-2.234	-7.371

	3x10m run - result	Standing broad jump - result	1 kg medicine ball throw - result	4-minute run - result	Total
$\bar{x}$	8.375	1.817	7.604	843.027	
**SD**	0.836	0.313	2.293	141.127	
**2%**	-1.738	-1.849	-1.570	-1.955	-6.291
**3%**	-1.643	-1.657	-1.460	-1.675	-5.802
**4%**	-1.523	-1.657	-1.351	-1.653	-5.356
**5%**	-1.500	-1.497	-1.307	-1.535	-5.015
**6%**	-1.404	-1.401	-1.264	-1.465	-4.765
**7%**	-1.368	-1.337	-1.220	-1.360	-4.553
**8%**	-1.285	-1.337	-1.176	-1.325	-4.320
**9%**	-1.261	-1.273	-1.132	-1.255	-4.126
**10%**	-1.165	-1.177	-1.132	-1.185	-3.986
**11%**	-1.165	-1.177	-1.089	-1.116	-3.790
**12%**	-1.118	-1.081	-1.045	-1.116	-3.658
**13%**	-1.058	-1.017	-1.001	-1.046	-3.498
**14%**	-1.046	-1.017	-0.958	-0.976	-3.355
**15%**	-1.020	-1.017	-0.958	-0.976	-3.224
**16%**	-0.939	-0.953	-0.914	-0.941	-3.118
**17%**	-0.927	-0.921	-0.892	-0.884	-2.973
**18%**	-0.915	-0.856	-0.870	-0.836	-2.873
**19%**	-0.855	-0.856	-0.826	-0.836	-2.763
**20%**	-0.807	-0.824	-0.783	-0.801	-2.653
**21%**	-0.807	-0.760	-0.783	-0.766	-2.564
**22%**	-0.760	-0.696	-0.739	-0.703	-2.464
**23%**	-0.712	-0.696	-0.695	-0.696	-2.357
**24%**	-0.688	-0.696	-0.695	-0.626	-2.259
**25%**	-0.688	-0.696	-0.695	-0.626	-2.164
**26%**	-0.640	-0.696	-0.695	-0.626	-2.061
**27%**	-0.605	-0.664	-0.651	-0.556	-1.957
**28%**	-0.569	-0.632	-0.608	-0.556	-1.864
**29%**	-0.569	-0.568	-0.608	-0.486	-1.784
**30%**	-0.569	-0.536	-0.608	-0.486	-1.712
**31%**	-0.533	-0.536	-0.564	-0.416	-1.629
**32%**	-0.485	-0.536	-0.520	-0.416	-1.543
**33%**	-0.449	-0.504	-0.520	-0.416	-1.454
**34%**	-0.449	-0.440	-0.476	-0.381	-1.383
**35%**	-0.449	-0.408	-0.476	-0.346	-1.318
**36%**	-0.426	-0.376	-0.476	-0.346	-1.247
**37%**	-0.390	-0.376	-0.433	-0.304	-1.162
**38%**	-0.342	-0.376	-0.433	-0.276	-1.105
**39%**	-0.330	-0.376	-0.389	-0.276	-1.030
**40%**	-0.330	-0.376	-0.345	-0.276	-0.953

	3x10m run - result	Standing broad jump - result	1 kg medicine ball throw - result	4-minute run - result	Total
$\bar{x}$	8.375	1.817	7.604	843.027	
**SD**	0.836	0.313	2.293	141.127	
**41%**	-0.318	-0.344	-0.345	-0.241	-0.896
**42%**	-0.282	-0.312	-0.323	-0.207	-0.817
**43%**	-0.247	-0.248	-0.301	-0.207	-0.735
**44%**	-0.211	-0.216	-0.258	-0.137	-0.661
**45%**	-0.211	-0.216	-0.258	-0.137	-0.597
**46%**	-0.211	-0.216	-0.258	-0.116	-0.530
**47%**	-0.187	-0.184	-0.258	-0.067	-0.454
**48%**	-0.151	-0.120	-0.214	-0.067	-0.366
**49%**	-0.103	-0.120	-0.170	0.003	-0.293
**50%**	-0.092	-0.056	-0.170	0.003	-0.215
**51%**	-0.092	-0.056	-0.148	0.003	-0.124
**52%**	-0.056	-0.056	-0.126	0.017	-0.052
**53%**	-0.020	-0.056	-0.083	0.073	0.023
**54%**	0.028	-0.056	-0.083	0.073	0.105
**55%**	0.028	-0.056	-0.039	0.073	0.188
**56%**	0.040	0.008	-0.039	0.143	0.256
**57%**	0.064	0.040	0.005	0.143	0.332
**58%**	0.099	0.104	0.040	0.178	0.424
**59%**	0.147	0.104	0.070	0.213	0.510
**60%**	0.147	0.104	0.092	0.213	0.581
**61%**	0.159	0.168	0.097	0.276	0.658
**62%**	0.195	0.200	0.136	0.283	0.752
**63%**	0.243	0.264	0.180	0.283	0.823
**64%**	0.266	0.264	0.180	0.353	0.919
**65%**	0.266	0.264	0.180	0.353	1.022
**66%**	0.290	0.264	0.223	0.388	1.116
**67%**	0.338	0.264	0.267	0.423	1.200
**68%**	0.386	0.328	0.267	0.423	1.305
**69%**	0.386	0.392	0.311	0.423	1.418
**70%**	0.410	0.424	0.355	0.428	1.531
**71%**	0.469	0.456	0.398	0.493	1.629
**72%**	0.505	0.520	0.420	0.528	1.731
**73%**	0.505	0.552	0.442	0.563	1.835
**74%**	0.529	0.584	0.530	0.582	1.989
**75%**	0.589	0.584	0.552	0.633	2.115
**76%**	0.624	0.584	0.573	0.668	2.227
**77%**	0.624	0.648	0.617	0.703	2.361
**78%**	0.696	0.712	0.617	0.772	2.487
**79%**	0.744	0.744	0.661	0.772	2.601

	3x10m run - result	Standing broad jump - result	1 kg medicine ball throw - result	4-minute run - result	Total
$\bar{x}$	8.375	1.817	7.604	843.027	
SD	0.836	0.313	2.293	141.127	
80%	0.744	0.840	0.705	0.807	2.753
81%	0.780	0.904	0.792	0.842	2.914
82%	0.863	0.904	0.836	0.874	3.046
83%	0.863	0.936	0.880	0.912	3.183
84%	0.911	1.033	0.967	0.982	3.336
85%	0.982	1.065	1.011	1.023	3.494
86%	0.994	1.161	1.055	1.052	3.665
87%	1.054	1.225	1.098	1.122	3.820
88%	1.102	1.225	1.164	1.122	4.033
89%	1.185	1.317	1.272	1.220	4.237
90%	1.233	1.385	1.317	1.262	4.471
91%	1.340	1.545	1.448	1.332	4.691
92%	1.412	1.545	1.492	1.472	4.958
93%	1.519	1.641	1.623	1.472	5.205
94%	1.651	1.767	1.798	1.681	5.476
95%	1.805	1.865	1.929	1.821	5.833
96%	1.913	1.929	2.148	1.821	6.240
97%	2.056	2.121	2.367	2.031	6.719
98%	2.295	2.281	2.717	2.241	7.382
99%	2.892	2.505	3.242	2.590	8.197
100%	9.216	4.106	4.554	3.569	12.839
